# Pediatric Hospitalizations for Unintentional Cannabis Poisonings and All-Cause Poisonings Associated With Edible Cannabis Product Legalization and Sales in Canada

**DOI:** 10.1001/jamahealthforum.2022.5041

**Published:** 2023-01-13

**Authors:** Daniel T. Myran, Peter Tanuseputro, Nathalie Auger, Lauren Konikoff, Robert Talarico, Yaron Finkelstein

**Affiliations:** 1 Clinical Epidemiology Program, Ottawa Hospital Research Institute, Ottawa, Ontario, Canada; 2 Department of Family Medicine, University of Ottawa, Ottawa, Ontario, Canada; 3 ICES uOttawa, Ottawa Hospital Research Institute, Ottawa, Ontario, Canada; 4 Department of Medicine, University of Ottawa, Ottawa, Ontario, Canada; 5 Bruyere Research Institute, University of Ottawa, Ottawa, Ontario, Canada; 6 University of Montreal Hospital Centre, Montreal, Quebec, Canada; 7 Institut national de santé publique du Quebec, Montreal, Quebec, Canada; 8 Department of Social and Preventive Medicine, School of Public Health, University of Montreal, Montreal, Quebec, Canada; 9 Department of Epidemiology, Biostatistics and Occupational Health, McGill University, Montreal, Quebec, Canada; 10 Divisions of Pediatric Emergency Medicine and Clinical Pharmacology and Toxicology, Hospital for Sick Children, Toronto, Ontario, Canada; 11 Departments of Paediatrics and Pharmacology and Toxicology, University of Toronto, Toronto, Ontario, Canada

## Abstract

**Importance:**

Canada legalized cannabis in October 2018 but initially prohibited the sale of edibles (eg, prepackaged candies). Starting in January 2020, some provinces permitted the sale of commercial cannabis edibles. The association of legalizing cannabis edibles with unintentional pediatric poisonings is uncertain.

**Objective:**

To evaluate changes in proportions of all-cause hospitalizations for poisoning due to cannabis in children during 3 legalization policy periods in Canada’s 4 most populous provinces (including 3.4 million children aged 0-9 years).

**Design, Setting, and Participants:**

This repeated cross-sectional study included all hospitalizations in children aged 0 to 9 years in Ontario, Alberta, British Columbia, and Quebec between January 1, 2015, and September 30, 2021.

**Exposures:**

Prelegalization (January 2015 to September 2018); period 1, in which dried flower only was legalized in all provinces (October 2018 to December 2019); and period 2, in which edibles were legalized in 3 provinces (exposed provinces) and restricted in 1 province (control province) (January 2020 to September 2021).

**Main Outcomes and Measures:**

The primary outcome was the proportion of hospitalizations due to cannabis poisoning out of all-cause poisoning hospitalizations. Data analysis was performed using descriptive statistics and Poisson regression models.

**Results:**

During the 7-year study period, there were 581 pediatric hospitalizations for cannabis poisoning (313 [53.9%] boys; 268 [46.1%] girls; mean [SD] age, 3.6 [2.5] years) and 4406 hospitalizations for all-cause poisonings. Of all-cause poisoning hospitalizations, the rate per 1000 due to cannabis poisoning before legalization was 57.42 in the exposed provinces and 38.50 in the control province. During period 1, the rate per 1000 poisoning hospitalizations increased to 149.71 in the exposed provinces (incidence rate ratio [IRR], 2.55; 95% CI, 1.88-3.46) and to 117.52 in the control province (IRR, 3.05; 95% CI, 1.82-5.11). During period 2, the rate per 1000 poisoning hospitalizations due to cannabis more than doubled to 318.04 in the exposed provinces (IRR, 2.16; 95% CI, 1.68-2.80) but remained similar at 137.93 in the control province (IRR, 1.18; 95% CI, 0.71-1.97).

**Conclusions and Relevance:**

This cross-sectional study found that following cannabis legalization, provinces that permitted edible cannabis sales experienced much larger increases in hospitalizations for unintentional pediatric poisonings than the province that prohibited cannabis edibles. In provinces with legal edibles, approximately one-third of pediatric hospitalizations for poisonings were due to cannabis. These findings suggest that restricting the sale of legal commercial edibles may be key to preventing pediatric poisonings after recreational cannabis legalization.

## Key Points

**Question:**

What is the association between legalization of recreational cannabis edibles and unintentional pediatric cannabis poisoning?

**Findings:**

This cross-sectional study of all children (n = 3.4 million) aged 0 to 9 years across 4 Canadian provinces found that jurisdictions that allowed the sale of cannabis edibles experienced much larger increases in cannabis poisonings and proportions of overall poisoning hospitalizations due to cannabis than the jurisdiction that prohibited edibles.

**Meaning:**

These findings suggest that restricting the sale of legal cannabis edibles may be a key policy to prevent unintentional pediatric cannabis poisonings following legalization.

## Teaser

This cross-sectional study uses population data to evaluate the association of pediatric hospitalizations for cannabis poisoning with legalization of edible cannabis products in Canada.

## Introduction

An increasing number of US states^1^ and countries around the world^2^ have legalized, or are considering legalizing, recreational cannabis. Discussions are ongoing in the US Senate to legalize cannabis federally. Mounting evidence suggests that legalizing recreational cannabis for adult use is associated with increases in pediatric cannabis poisonings. Young children with cannabis poisoning frequently present with high-acuity conditions, including a decreased level of consciousness, respiratory depression, and seizures.^3,4,5^ Previous studies from Colorado, Washington, and Massachusetts reported that legalization of recreational^5,6^ and medical cannabis^7^ was associated with increases in cannabis-related calls to poison control centers and emergency department (ED) visits for cannabis poisonings in children. Recent studies from Canada found that ED visits and hospitalizations for cannabis poisonings in children increased after legalization of recreational cannabis, particularly when legal cannabis edibles were introduced.^8^ However, the introduction of edible cannabis in Canada coincided closely with the COVID-19 pandemic when there were large changes in pediatric health care visit patterns, including reports of increases in pediatric poisoning events.^9,10^ Consequently, whether pediatric poisonings have increased primarily as a result of the legalization of recreational cannabis in general, an increase in children’s access to ready-to-consume commercially produced legal cannabis edibles, COVID-19–related factors, or a combination of factors is unclear. Also unclear is how temporal trends in cannabis poisonings differ from all-cause pediatric poisonings and the relative burden of cannabis on poisoning events in children.

In October 2018, Canada legalized the sale and use of nonmedical, or recreational, cannabis to adults (age ≥18 years) at the national level. Canada took a unique, 2-phase approach to legalizing recreational cannabis. Initially, Canada allowed only the sale of dried cannabis flower, while later permitting provinces to sell a wider variety of cannabis products, including edibles (eg, tetrahydrocannabinol [THC]-containing gummies, candies, baked goods, and chocolates), concentrates, and THC-infused beverages.^11^ While all provinces in Canada were legally required to allow the sale of dried cannabis flower in October 2018, they could choose to allow or prohibit the sale of the expanded commercial edible products. In Ontario, British Columbia, and Alberta (Canada’s first, third, and fourth most populous provinces, respectively, with a combined population of 24.3 million in 2020), a wide variety of cannabis edibles and THC-infused beverages came to market in January 2020.^12,13^ In contrast, Quebec (Canada’s second most populous province with a population of 8.6 million in 2020) prohibited the sale of sweets, candies, desserts and chocolates, and products that could be deemed potentially attractive to children and young adults.^14^ The effect of this decision was that no edible products other than THC-infused beverages came to market in Quebec during our study period. Preliminary observations from our team found larger increases in hospitalizations due to cannabis in children aged 0 to 9 years in jurisdictions that allowed the sale of edibles after legalization.^15^

The purpose of this study was to leverage the universal but staggered rollout of recreational cannabis in Canada (eg, dried flower only, then expanded products) with differing policy approaches to edibles among provinces in order to ascertain the unique effects of legalization with vs without edibles. We contextualize our findings within temporal patterns of hospitalizations for pediatric cannabis poisonings compared with changes in overall pediatric hospitalizations and all-cause pediatric poisoning hospitalizations to account for COVID-19 effects and ascertain the overall burden of cannabis on hospitalizations for pediatric poisonings.

## Methods

This cross-sectional study was approved by the Ottawa Hospital Research Institute’s Research Ethics Board. Because this study used deidentified aggregate health information, no informed consent was required. This study followed the Strengthening the Reporting of Observational Studies in Epidemiology (STROBE) reporting guideline.

### Study Design

We conducted a repeated, cross-sectional, population-based study of all hospitalizations in children aged 0 to 9 years in Canada’s 4 most populous provinces (Ontario, Quebec, Alberta, and British Columbia, totaling 32.9 million residents [86% of the Canadian population]) between January 1, 2015, and September 30, 2021, using routinely collected hospital discharge records. Data on race and ethnicity were not available in the data sets used in this study. We divided the study period into 3 policy periods: prelegalization (January 2016 to September 2018); legalization of dried flower, seeds, and oil only in all provinces (period 1, October 2018 to December 2019); and legalization with commercial cannabis edibles permitted in 3 provinces (Ontario, Alberta, and British Columbia [exposed provinces]) and restricted in 1 province (Quebec [control province]) (period 2, January 2020 to September 2021). Period 2 also overlapped with the COVID-19 pandemic. Data were obtained from hospital records from each province’s universal health care insurance, which captures all residents of Canada.

### Data Sources

We obtained hospitalization data from the Canadian Institutes for Health Information (CIHI) and Régie de l'assurance maladie du Québec (RAMQ). The CIHI Discharge Abstract Database captures hospitalization admission for Ontario, Alberta, and British Columbia, and the RAMQ Maintenance and Use of Data for the Study of Hospital Clientele database captures hospitalization admissions for Quebec. We combined data for British Columbia and Alberta to minimize data suppression (ie, privacy rules from CIHI require that cells with fewer than 5 events be suppressed). We obtained the monthly value (in Canadian dollars [CAD$]) of legal cannabis sales in each province from Statistics Canada. Every licensed cannabis retailer in Canada is required under the Cannabis Act to report all physical and online sales each month, and these sales represent a census of all legal sales.^16^

### Outcomes

The primary outcome was the proportion of hospitalizations due to cannabis poisoning out of all-cause poisoning hospitalizations. We focused on pediatric cannabis poisonings that led to hospitalization, as those generally represent more serious exposures requiring inpatient care and with potential longitudinal implications. We identified cannabis poisoning hospitalizations when 1 of the following *International Statistical Classification of Diseases and Related Health Problems, 10th revision* (*ICD-10*) codes was listed as the main or contributing reason for hospitalization: T40.7 (poisonings by cannabis, including derivatives) or F12.X (mental and behavioral disorders due to use of cannabinoids). We identified hospitalizations for all-cause poisonings (ie, due to pharmaceuticals and nonpharmaceuticals) when the main or contributing reason for hospitalization was coded T36 to T65 in the *ICD-10*. As a secondary outcome, we evaluated the proportion of hospitalizations due to cannabis poisonings out of the total all-cause hospitalizations in the studied age group. The purpose of these outcomes was to evaluate the overall burden of cannabis poisonings and to contextualize the results amid changes in health-seeking behaviors during the COVID-19 pandemic (eg, reduced all-cause pediatric ED visits^9^) and the association with public health measures that kept children at home more often (eg, school and daycare closures).

### Statistical Analysis

We present descriptive statistics, including means and proportions, to characterize and compare hospitalizations due to cannabis poisonings across the 3 regulatory periods and the whole study period. We obtained incidence rate ratios (IRRs) using saturated Poisson regression models. Our saturated Poisson models included the count of events in each jurisdiction over the 3 policy periods (prelegalization, legalization period 1, and legalization period 2), an indicator variable for each jurisdiction, and 3 level categorical variables indicating the different policy periods. We included an interaction term between the jurisdictions. We offset our models by the log-transformed count of total hospitalizations for pharmaceutical and nonpharmaceutical poisonings (including cannabis) or total all-cause hospitalizations. We estimated IRRs and 95% CIs for each legalization period by exponentiating coefficient estimates from the model fit. Data analyses were conducted using SAS, version 9.4 software (SAS Institute Inc).

## Results

During the 7-year study period, there were 581 hospitalizations for pediatric poisoning due to cannabis. The mean (SD) age at the time of hospitalization was 3.6 (2.5) years, and 53.9% were male and 46.1% female. Table 1 lists the characteristics of hospitalizations due to cannabis poisonings in children aged 0 to 9 years prelegalization, after cannabis legalization (period 1), and after the legalization of commercial edible products (period 2) by jurisdiction. The proportion of males was similar over the 3 periods, while the mean (SD) age increased from 3.0 (2.7) years prelegalization to 3.9 (2.5) years in period 2. Of the 581 hospitalizations for cannabis poisoning, 120 occurred prelegalization (31 months), 105 occurred during legalization period 1 (14 months), and 356 occurred during legalization period 2 (19 months).

**Table 1.** Child and Hospitalization Characteristics by Cannabis Legalization Period

	No. (%)			
	Entire study period (Jan 2015-Sept 2021)	Prelegalization (Jan 2015-Sept 2018)	Legalization	
			Period 1 (Oct 2018-Dec 2019)	Period 2 (Jan 2020-Sept 2021)
Type of hospitalization, No.				
All-cause	3 143 673	1 807 329	594 990	741 354
All-cause poisonings	4406	2347	759	1300
Cannabis poisonings	581	120	105	356
Characteristics of children hospitalized for cannabis poisoning				
Age, mean (SD), y	3.6 (2.5)	3.0 (2.7)	3.1 (2.3)	3.9 (2.5)
Sex				
Male	313 (53.9)	64 (53.3)	56 (53.3)	193 (54.2)
Female	268 (46.1)	56 (46.7)	49 (46.7)	163 (46.8)
Province (population aged 0-9 y, 2018)				
Quebec (900 843)	102 (17.6)	31 (25.8)	27 (25.7)	44 (12.4)
Ontario (1 481 381)	285 (49.1)	44 (36.7)	36 (34.3)	205 (57.6)
Alberta and British Columbia (1 020 468)	194 (33.4)	45 (37.5)	42 (40.0)	107 (30.1)

**Figure 1.**
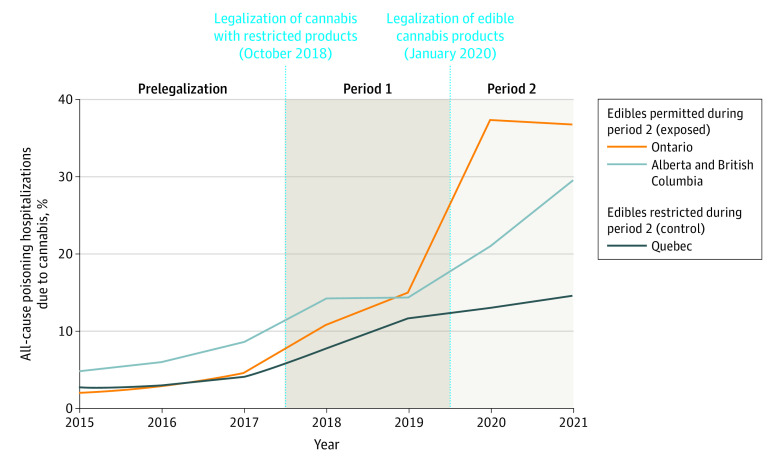
Percentage of All-Cause Poisoning Hospitalizations in Children Aged 0 to 9 Years Due to Cannabis in Ontario, Alberta, British Columbia, and Quebec

The proportion of poisoning hospitalizations due to cannabis out of the total poisoning hospitalizations increased 844.3% over the 7-year study period from 20 of 651 (3.1%) in 2015 to 179 of 617 (29.0%) in 2021. The proportion of total poisoning hospitalizations due to cannabis increased in all provinces after legalization of dried flower only (period 1) and further increased in exposed provinces that permitted sales of edibles but not in the control province during period 2 (Figure 1). Similar findings were observed in the proportion of hospitalizations due to cannabis out of all-cause hospitalizations(eFigure 1 in Supplement 1).

Before legalization, the proportion of poisoning hospitalizations due to cannabis out of all pediatric poisoning hospitalizations ranged from 31 of 808 (3.8%) in Quebec to 45 of 643 (7.0%) in Alberta and British Columbia. During period 1, the rate per 1000 poisoning hospitalizations due to cannabis had a similar increase across the studied provinces (IRR, 2.71; 95% CI, 2.06-3.55), increasing 2.6-fold in the exposed provinces (from 57.42 to 149.71) and 3.1-fold in the control province (from 38.50 to 117.52). During period 2 the rate per 1000 poisoning hospitalizations due to cannabis in exposed provinces (318.04) increased an additional 2.2-fold compared with period 1 (IRR, 2.16; 95% CI, 1.68-2.80). In contrast, the rate per 1000 poisoning hospitalizations in the control province (137.93) did not change during period 2 compared with period 1 (IRR, 1.18; 95% CI, 0.71-1.97) (Table 2).

**Table 2.** Rate of Hospitalizations Due to Cannabis Poisoning by Legalization Period

Geographic region	Entire study period (Jan 2015-Sept 2021)	Prelegalization (Jan 2015-Sept 2018)	Legalization		IRR (95% CI)	
			Period 1 (Oct 2018-Dec 2019)	Period 2 (Jan 2020-Sept 2021)	Period 1 vs prelegalization	Period 2 vs period 1
**Rate per 1000 poisoning hospitalizations**						
All provinces	131.87	50.89	140.37	273.85	2.71 (2.06-3.55)	1.98 (1.59-2.49)
Exposed provinces: Ontario, Alberta, British Columbia	156.95	57.42	149.71	318.04	2.55 (1.88-3.46)	2.16 (1.68-2.80)
Ontario	164.74	48.51	133.82	370.04	2.73 (1.76-4.24)	2.79 (1.95-4.09)
Alberta and British Columbia	146.75	69.98	166.67	250.59	2.30 (1.51-3.51)	1.54 (1.07-2.26)
Control province: Quebec	75.33	38.50	117.52	137.93	3.05 (1.82-5.11)	1.18 (0.71-1.97)
**Rate per 1000 all-cause hospitalizations**						
All provinces	0.18	0.07	0.18	0.48	2.57 (2.03-3.48)	2.72 (2.18-3.42)
Exposed provinces: Ontario, Alberta, British Columbia	0.22	0.07	0.19	0.61	2.38 (1.75-3.24)	3.14 (2.44-4.08)
Ontario	0.22	0.06	0.15	0.66	2.42 (1.52-3.85)	4.48 (3.13-6.57)
Alberta and British Columbia	0.23	0.09	0.27	0.53	2.91 (1.86-4.53)	1.99 (1.38-2.92)
Control province: Quebec	0.10	0.05	0.14	0.19	2.67 (1.54-4.63)	1.37 (0.83-2.31)

**Figure 2.**
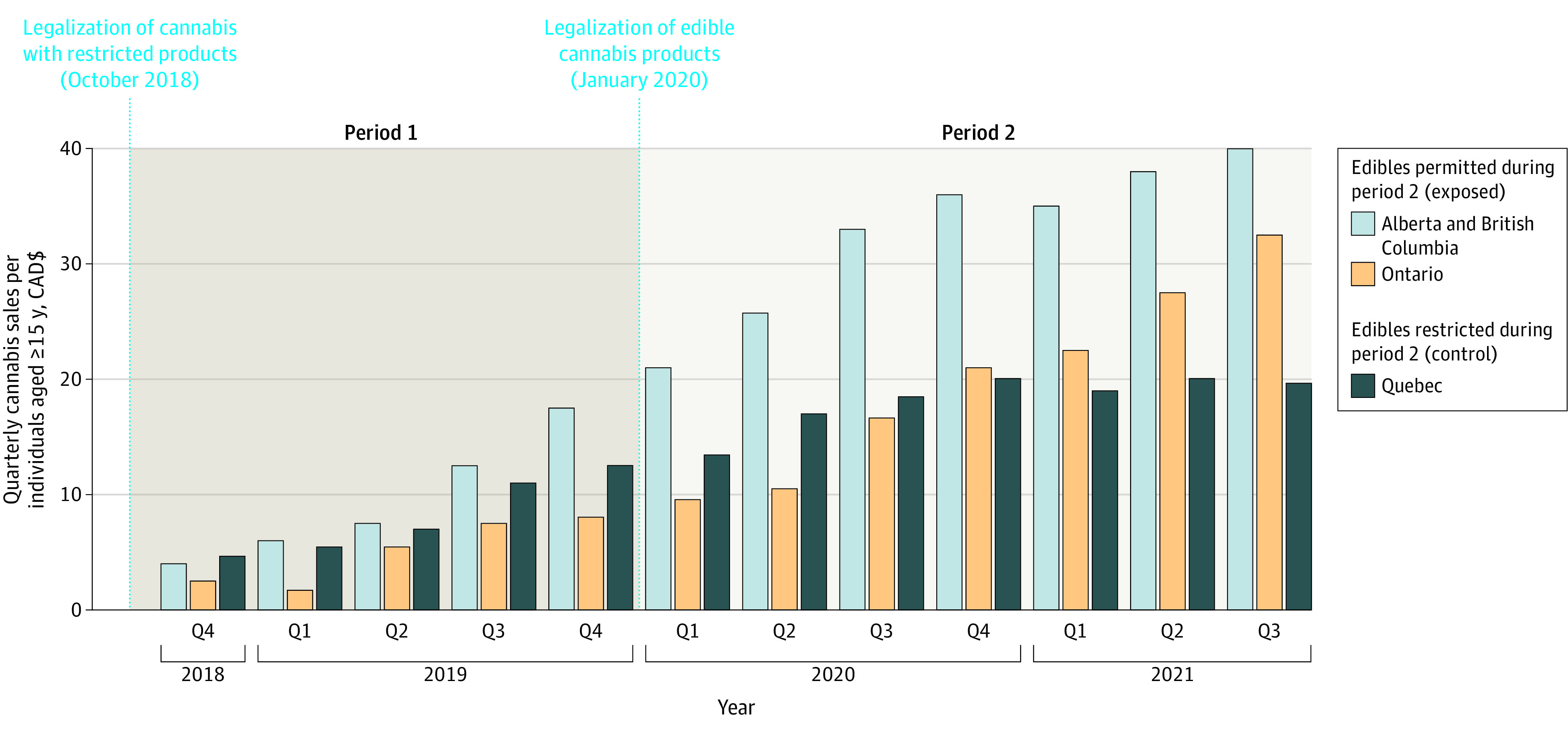
Quarterly Cannabis Sales per Individual Between October 2018 and September 2021 CAD$ indicates Canadian dollars; Q, quarter.

Legal per capita cannabis sales increased in all provinces over the study period (Figure 2). During period 1, the mean (SD) quarterly per capita sales (CAD$ per individual aged ≥15 years) were $5.14 ($2.86) in Ontario, $8.03 ($3.48) in Quebec, and $9.75 ($5.47) in Alberta and British Columbia. During period 2, the mean (SD) quarterly per capita sales were similar in Ontario ($19.87 [$8.31]) and Quebec ($18.55 [$2.64]) and were highest in Alberta and British Columbia ($33.15 [$6.78]). Sales data for cannabis edibles were only publicly available for the province of Ontario, and sales (CAD$ per individual aged ≥15 years) increased by 390.0% between quarter 1 of 2020 ($0.30) and quarter 3 of 2021 ($1.47) (eFigure 2 in Supplement 1).

## Discussion

In this repeated cross-sectional study of more than 3.4 million children aged 0 to 9 years between 2015 and 2021, we found that recreational cannabis legalization was associated with a large increase (from 3.1% in 2015 to 29.0% in 2021) in unintentional cannabis poisoning hospitalizations in young children and their relative burden on the health care system. These findings suggest that cannabis is now a leading cause of pediatric poisoning hospitalizations in Canada. Allowing the sale of legal commercial cannabis edibles appears to be a key driver of these trends. Shortly following legalization in Canada, when the sale of edibles was prohibited, all studied provinces experienced a similar 2.6- to 3.1-fold increase in the rate per 1000 hospitalizations due to cannabis poisoning. During the period that edibles were permitted, the 3 provinces that introduced commercial edibles experienced a further 2.2-fold increase in this rate, while the province that prohibited their sale saw no further increase. The overall increases in unintentional pediatric poisonings occurred despite regulations by Canadian authorities aimed at reducing the incidence and severity of unintentional pediatric poisonings, including a maximum of 10 mg THC per edible package (representing a 10-fold smaller amount than allowed in several US jurisdictions), plain and child-resistant packaging requirements, and consumer education campaigns.^8,16^

Prior research from states that have legalized or decriminalized adult cannabis use have reported increases in cannabis poisonings in children. A tertiary care hospital in Colorado found that the rate of cannabis-related ED visits in children aged 0 to 9 years doubled from the 2 years before legalization to the 2 years after legalization.^5^ A study among poison control centers across the US between 2005 and 2011 found that calls for cannabis-related visits in children aged 0 to 9 years increased by 30.3% per year in states that had decriminalized cannabis vs 1.5% per year in states where cannabis remained illegal.^17^ Another study of poison control center calls for individuals of all ages across the US reported a 33.6% increase in total calls regarding cannabis between 2017 and 2019 and a 347.9% increase in calls regarding exposure to cannabis edibles or concentrates.^18^

The Canadian experience of cannabis legalization, with marked differences in rates of hospitalized children for cannabis poisoning between the provinces that allowed the sale of commercial edibles and the province that prohibited it, offers key insights to inform policy makers in states and countries considering legalizing adult recreational cannabis use and to those reviewing their currently enacted policies. These findings are particularly relevant and timely in the context of the recent discourse in the US and other countries regarding legalization of recreational cannabis use on a federal level. Our findings of disparate proportions of pediatric poisonings in Quebec and Ontario, despite the comparable overall dollar value of legal cannabis sales during the study, suggest that product type rather than sales volume is a key factor associated with pediatric poisonings. The large increase in harms associated with the sale of commercial cannabis edibles and concentrates in exposed jurisdictions suggests that restricting or prohibiting the sale of these products could be a highly effective regulatory act to reduce the frequency and severity of unintentional pediatric poisonings. Another important policy consideration is child-resistant packaging. Child-resistant packaging, mandated by the US 1970 Poison Prevention Packaging Act, is highly effective and has resulted in substantial decreases in pediatric injuries and deaths from pharmaceutical and nonpharmaceutical substances.^19^ While mandating child-resistant packaging for cannabis products in Canada likely prevented even larger increases in cannabis poisonings, our findings caution that this measure alone may be insufficient. Other jurisdictions could also consider requiring unit-dose (blister) packaging, a particularly effective form of child-resistant packaging for multidose cannabis products.^19^

### Strengths and Limitations

Our study has several strengths. First, it includes the use of population-level data, which capture all hospitalizations for 86% of children in Canada. Second, we evaluated the associations of regulatory policies with pediatric cannabis poisonings by using universal and staggered rollout data of cannabis legalization (ie, initially flower products only followed by commercial edibles) and the provincial variations in permitting the sale of edibles (ie, prohibited in Quebec, allowed elsewhere). Third, the large increase in cannabis poisoning hospitalizations as a proportion of all-cause poisoning hospitalizations suggests alternative explanations of all-cause poisoning trends (eg, cannabis hospitalizations have increased because hospitalizations for poisonings in general have increased over the cannabis legalization periods).

Our study also has several limitations. First, hospitalizations did not include data on the cannabis source (eg, illicit vs legal). However, the abrupt increase in pediatric hospitalizations in the 3 provinces that allowed the legal sale of edibles, contrasted with no increase in the province that prohibited legal edibles sales, argues against a major contribution of illegal products to the observed trends. Second, there was an overlap of the COVID-19 pandemic with the period cannabis edibles appeared on the retail market. However, the provinces under study had similar numbers of COVID-19 cases (eg, 52.0 vs 56.7 cases per 1000 individuals in the exposed provinces and the control province, respectively) and public health measures,^20,21^ suggesting that pandemic influences do not explain the observed differences in changes in pediatric poisoning hospitalization rates by province. Third, national cannabis sales were only available in dollars rather than by product volumes (eg, milligrams of THC) or sales types (eg, flower vs edible products). Importantly, while there were minor taxation and pricing differences among provinces and over time (eg, the price of legal flower products gradually declined over time but new products, such as concentrates and vapes, were more expensive), the value of cannabis sold is likely broadly reflective of the volume of cannabis sold.^22^

## Conclusions

In this cross-sectional study, there were large increases in hospitalizations for unintentional cannabis poisonings among children following legalization of recreational cannabis use by adults. Most of this increase was associated with the introduction of legal, commercial edible cannabis products and occurred despite strict regulatory and educational measures aimed at reducing cannabis poisonings in young children. Our findings suggest that placing restrictions on the sale of visually attractive and palatable commercial cannabis edibles is a key strategy and policy consideration for preventing unintentional pediatric cannabis poisonings for the US and other countries considering legalization of recreational cannabis.

## Notes

Supplement 1. **eFigure 1.** Percentage of All-Cause Poisoning Hospitalizations in Children Aged 0 to 9 Years Due to Cannabis in Ontario, Alberta, British Columbia, and Quebec **eFigure 2.** Quarterly Edible Cannabis Sales in Ontario, Canada, in Canadian Dollars Per Individual Between October 2018 and September 2021

Supplement 2.
Data Sharing Statement
