# Supplementary material for: Pediatric Hospitalizations for Unintentional Cannabis Poisonings and All-Cause Poisonings Associated With Edible Cannabis Product Legalization and Sales in Canada
Source: JAMA Health Forum. 2023 Jan 13;4(1):e225041. doi: 10.1001/jamahealthforum.2022.5041 (PMC9857209; doi:10.1001/jamahealthforum.2022.5041)
Supplement: Supplement 1. — eFigure 1. Percentage of All-Cause Poisoning Hospitalizations in Children Aged 0 to 9 Years Due to Cannabis in Ontario, Alberta, British Columbia, and Quebec eFigure 2. Quarterly Edible Cannabis Sales in Ontario, Canada, in Canadian Dollars Per Individual Between October 2018 and September 2021 [file jamahealthforum-e225041-s001.pdf]

Supplemental Online Content

Myran DT, Tanuseputro P, Auger N, Konikoff L, Talarico R, Finkelstein Y. Pediatric hospitalizations for unintentional cannabis poisonings and all-cause poisonings associated with edible cannabis product legalization and sales in Canada. *JAMA Health Forum*. 2023;4(1):e225041. doi:10.1001/jamahealthforum.2022.5041

eFigure 1. Percentage of All-Cause Poisoning Hospitalizations in Children Aged 0 to 9 Years Due to Cannabis in Ontario, Alberta, British Columbia, and Quebec
eFigure 2. Quarterly Edible Cannabis Sales in Ontario, Canada, in Canadian Dollars Per Individual Between October 2018 and September 2021

This supplemental material has been provided by the authors to give readers additional information about their work.

© 2023 Myran DT et al. *JAMA Health Forum.*

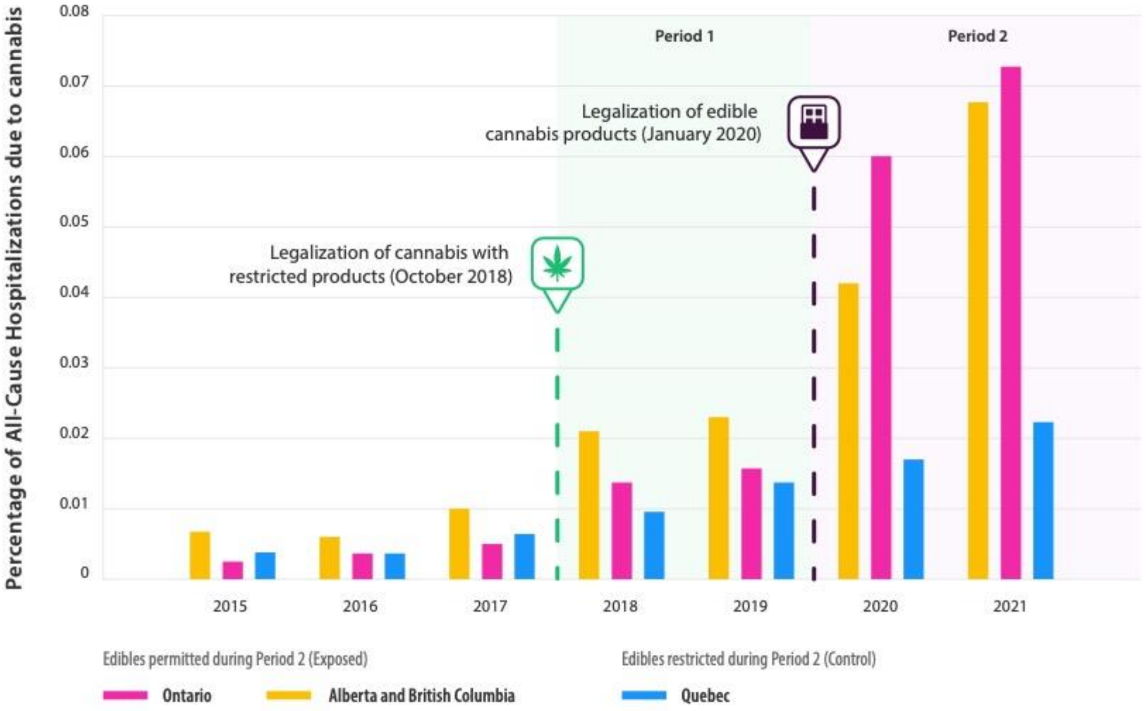

eFigure 1. Percentage of All-Cause Poisoning Hospitalizations in Children Aged 0 to 9 Years Due to Cannabis in Ontario, Alberta, British Columbia, and Quebec

© 2023 Myran DT et al. *JAMA Health Forum.*

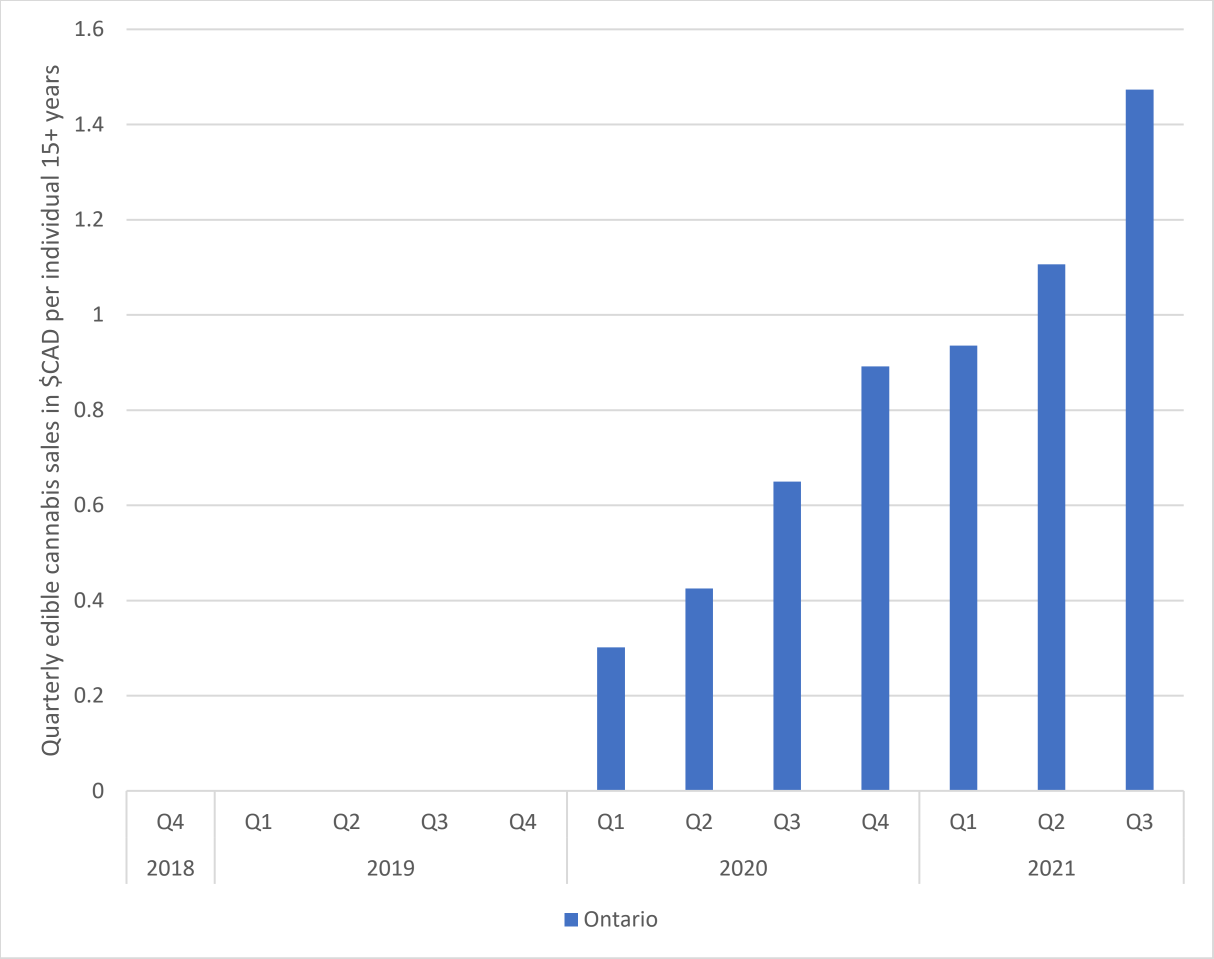

eFigure 2. Quarterly Edible Cannabis Sales in Ontario, Canada, in Canadian Dollars Per Individual Between October 2018 and September 2021

Ontario edible cannabis sales were obtained from quarterly reviews from the Ontario Cannabis Store https://ocs.ca/pages/insights-publication.

© 2023 Myran DT et al. *JAMA Health Forum.*
