# Supplementary material for: Pediatric Hospitalizations for Unintentional Cannabis Poisonings and All-Cause Poisonings Associated With Edible Cannabis Product Legalization and Sales in Canada
Source: JAMA Health Forum. 2023 Jan 13;4(1):e225041. doi: 10.1001/jamahealthforum.2022.5041 (PMC9857209; doi:10.1001/jamahealthforum.2022.5041)
Supplement: Supplement 2. — Data Sharing Statement [file jamahealthforum-e225041-s002.pdf]

Data Sharing Statement

Myran. Pediatric Hospitalizations for Unintentional Cannabis Poisonings and All-Cause Poisonings Associated With Edible Cannabis Product Legalization and Sales in Canada. *JAMA Health Forum*. Published January 13, 2023. doi:10.1001/jamahealthforum.2022.5041

Data
Data available: No
